# Early-Onset Colorectal Cancer: Clinicopathological Features and Surgical Outcomes in Patients Treated with Curative Intent at a Tertiary Center

**DOI:** 10.3390/cancers18121934

**Published:** 2026-06-14

**Authors:** Clemente Junior Nappi, Arturo Cirera de Tudela, Marc Martí-Gallostra, Miquel Kraft Carre, José Perea, Eloy Espín-Basany

**Affiliations:** 1 Colorectal Surgery, Department of General and Digestive Surgery, Vall d’Hebron University Hospital, 08035 Barcelona, Spain; nappiclementejunior@gmail.com (C.J.N.);; 2 Department of Surgery, Universitat Autònoma de Barcelona (UAB), 08193 Barcelona, Spain; 3 Surgery Department, Vithas Arturo Soria University Hospital, 28043 Madrid, Spain; 4 Molecular Medicine Unit, Department of Medicine, Biomedical Research Institute of Salamanca (IBSAL), Institute of Molecular and Cellular Biology of Cancer (IBMCC), University of Salamanca-SACYLCSIC, 37007 Salamanca, Spain

**Keywords:** early-onset colorectal cancer, EOCRC, minimally invasive surgery, laparoscopic surgery, robotic surgery, clinicopathological features, surgical outcomes, colorectal cancer

## Abstract

**Background:** The incidence of early-onset colorectal cancer (EOCRC) has increased worldwide and now represents approximately 10% of colorectal cancers in high-income countries. EOCRC is frequently associated with advanced pathological features, although its clinical behavior and optimal management remain incompletely defined. **Methods:** We performed a retrospective single-center study including 88 consecutive patients aged ≤50 years who underwent curative-intent colorectal cancer resection between January 2019 and December 2023 at a tertiary referral center. Perioperative outcomes, pathological characteristics, and recurrence patterns were analyzed. **Results:** The median age was 44 years, and 67% of tumors were located in the colon. Pathological nodal involvement (pN+) was observed in 47.7% of patients, with a high prevalence of adverse features including perineural invasion (62.5%), tumor budding (17.0%), and tumor deposits (15.9%). Minimally invasive surgery was performed in 79% of patients and was associated with shorter hospital stay without increased postoperative morbidity (19.3%). During a median follow-up of 31.3 months [IQR 21.6–44.6], recurrence occurred in 40 patients (45.5%) and was predominantly distant (75.0%). Among patients with recurrence, 21 (52.5%) underwent surgical reintervention, most commonly hepatic and pulmonary resections. Rectal cancer was associated with higher rates of stoma formation and major postoperative complications compared to colon cancer. **Conclusions:** EOCRC is characterized by a high prevalence of adverse pathological features and a substantial rate of distant recurrence. However, a relevant proportion of recurrences remains amenable to surgical treatment in selected patients. Management in a specialized tertiary center allows achievement of high-quality surgical outcomes and supports an aggressive multidisciplinary approach.

## Simple Summary

Early-onset colorectal cancer, defined as colorectal cancer diagnosed before the age of 50 years, is increasing worldwide and represents an important clinical challenge. In this study, we analyzed 88 young patients who underwent curative-intent surgery for colorectal cancer at a high-volume tertiary referral center. Many patients presented with adverse pathological features, including lymph node involvement, perineural invasion, tumor budding, and tumor deposits. Recurrence was frequent and was mainly distant, rather than isolated local relapse. However, more than half of the patients who developed recurrence underwent further surgical treatment, most commonly for liver or lung metastases. These findings suggest that early-onset colorectal cancer often behaves aggressively, but also that selected patients may benefit from active multidisciplinary management and repeated surgical treatment when recurrence remains potentially resectable.

## 1. Introduction

Early-onset colorectal cancer (EOCRC), defined as colorectal cancer diagnosed before the age of 50 years, has demonstrated a consistent and concerning increase in incidence worldwide over the past two decades [1,2,3,4,5,6,7,8,9,10,11,12,13,14,15,16,17,18,19,20]. While overall colorectal cancer mortality has declined—largely due to population-based screening programs and advances in multimodal therapy—the incidence of EOCRC has nearly doubled in several Western countries, contrasting with the downward trend observed in late-onset disease [4,5]. Currently, EOCRC accounts for approximately 10% of all newly diagnosed colorectal cancer cases in high-income regions [6,7].

The causes underlying this epidemiologic shift remain incompletely understood. Proposed contributing factors include Westernized dietary patterns, obesity, physical inactivity, alterations in the gut microbiome, antibiotic exposure, environmental pollution, and hereditary cancer predisposition syndromes [6,8,9]. Although pathogenic germline variants are identified in up to 16–25% of patients with EOCRC, most commonly involving mismatch repair (MMR) genes such as MLH1, MSH2, MSH6, and PMS2, the majority of EOCRC cases occur sporadically [6,8,9,10,11,12,13]. From a clinical perspective, EOCRC is frequently associated with more advanced stage at diagnosis, including higher rates of nodal involvement and distant metastases [14,15,16,17,18,19,20,21,22,23]. Tumors are more often located in the distal colon and rectum and may display adverse pathological features, such as poor differentiation and signet-ring cell histology [1,2,3,15]. These findings suggest a potentially more aggressive disease phenotype and highlight the need for improved characterization of this patient population [19,20].

In order to harmonize the assessment and management of early-onset colorectal cancer and to provide reliable and up-to-date epidemiological and clinical data, several collaborative initiatives have been established. For example, the Spanish Early-onset Colorectal Cancer Cohort (SECOC) [1] is a multicenter study designed to comprehensively characterize the molecular landscape of EOCRC in Spain, including both somatic and germline alterations. Data from SECOC have contributed to large European analyses within the GEOCODE (Global Early-Onset Colorectal Cancer Database) consortium, where most tumors were found to be mismatch repair proficient, while a smaller proportion exhibited deficient MMR, with Lynch syndrome identified in a relevant subset of tested patients [24,25].

This initiative brings together multiple institutions worldwide to collect standardized clinical, pathological, and outcome data in patients diagnosed with EOCRC, thereby facilitating large-scale analyses and improving the quality of evidence available for this emerging clinical entity. The Hospital Universitari Vall d’Hebron (HUVH) participates in this collaborative network, contributing institutional data to the shared registry.

Within this framework, the present study aims to evaluate the clinicopathological characteristics, surgical management, and short-term oncological outcomes of patients with EOCRC undergoing curative-intent resection at a high-volume tertiary referral center.

## 2. Materials and Methods

This retrospective single-center cohort study included consecutive patients aged ≤50 years who underwent curative-intent colorectal resection between January 2019 and December 2023 at a high-volume tertiary referral center (Hospital Universitari Vall d’Hebron, Barcelona, Spain). The clinical cohort analyzed in this study was derived exclusively from Hospital Universitari Vall d’Hebron.

Although consecutive patient inclusion was used to minimize selection bias, the cohort should not be considered population-based, as it reflects the activity and referral patterns of a tertiary referral center. This study complied with the Strengthening the Reporting of OBservational studies in Epidemiology (STROBE) recommendations [26].

### 2.1. Inclusion and Exclusion Criteria

All consecutive patients aged ≤50 years who underwent curative-intent colorectal resection for histologically confirmed colorectal cancer were screened for eligibility. Patients with synchronous metastatic disease were included only when the overall treatment strategy was considered potentially curative after multidisciplinary team evaluation. This included selected patients undergoing primary tumor resection within a curative-intent or conversion strategy, with systemic treatment and/or local treatment of metastatic disease when appropriate. Patients undergoing exclusively palliative or non-curative procedures were excluded. Only patients operated on at the Colorectal Surgery Unit of Vall d’Hebron University Hospital with complete demographic, clinical, surgical, pathological, and follow-up data available in the institutional database were included in the analysis.

Patients with incomplete clinical records or those who underwent non-curative procedures were excluded. As a retrospective single-center study, potential sources of bias include selection bias and limited generalizability. Consecutive patient inclusion was used to minimize selection bias.

### 2.2. Study Aims

The primary aim of the study was to describe the clinicopathological characteristics and surgical outcomes of patients with early-onset colorectal cancer undergoing curative-intent resection at a tertiary referral center.

Secondary aims included comparing clinicopathological features between colon and rectal cancers; evaluating differences between age subgroups (<40 vs. 40–50 years); assessing postoperative morbidity according to surgical approach; and analyzing pathological high-risk features and short-term oncologic outcomes in this patient population.

### 2.3. Definitions and Data of Interest

Demographic, endoscopic, surgical, pathological, and oncologic data were extracted from a prospectively maintained institutional database.

Tumor location was categorized according to anatomical site and further grouped as colon or rectum for comparative analyses. Distance from the anal verge and tumor length were obtained from endoscopic reports. Tumor circumferential involvement was recorded as the percentage of bowel circumference affected during the endoscopy.

In our cohort, mismatch repair (MMR) status was routinely assessed using immunohistochemistry for MLH1, MSH2, MSH6, and PMS2 proteins, according to institutional protocols. Microsatellite instability (MSI) testing and extended molecular analyses (including RAS mutations) were not systematically performed in all patients but were conducted based on multidisciplinary discussion.

Postoperative complications occurring during the index hospitalization were graded according to the Clavien–Dindo classification, and the highest recorded grade per patient was considered for analysis. Complications after discharge occurring within 90 days were additionally captured and analyzed as a binary outcome (yes/no).

Surgical approach was categorized as open, laparoscopic, or robotic. Minimally invasive surgery included laparoscopic and robotic procedures. Surgical procedures were also classified as elective or emergency. The choice of surgical approach was determined by tumor location, disease extent, emergency versus elective setting, previous abdominal surgery, patient-related factors, and surgeon expertise. Open surgery was generally reserved for emergency cases, locally advanced tumors, or cases in which minimally invasive surgery was considered unsuitable. Robotic surgery was preferentially used for selected rectal and technically demanding pelvic procedures when available.

Recurrence was classified as local, locoregional, or distant. Local recurrence was defined as recurrence at the anastomotic site or within the bowel wall. Locoregional recurrence was defined as recurrence involving the tumor bed, pelvis/mesorectum, or regional lymph nodes. Distant recurrence included liver, lung, peritoneal, distant nodal, bone, brain, or other extra-regional disease. Peritoneal recurrence was classified as distant recurrence. Combined patterns including distant disease were classified as distant recurrence.

### 2.4. Statistical Analysis

Continuous variables are presented as median (interquartile range), categorical variables are reported as number (percentage). Comparisons between groups were performed using non-parametric tests for continuous variables and chi-square or Fisher’s exact tests for categorical variables. A two-sided *p*-value < 0.05 was considered statistically significant.

## 3. Results

### 3.1. Overall Cohort Characteristics

Between 2019 and 2023, a total of 125 patients younger than 50 years with suspected or confirmed colorectal neoplasia were registered in the institutional database. Of these, 88 patients met the inclusion criteria and were included in the final analysis. Thirty-seven patients were excluded: 4 underwent surgery at another institution, 5 did not undergo surgery because of disease progression, and 28 were excluded because of incomplete clinical data or incomplete follow-up. The median age at the time of surgery was 44 years, with a balanced sex distribution. The demographic characteristics of the cohort are detailed in Table 1 Surgical outcomes for the entire cohort are summarized in Table 2.

Stage IV disease was present in 30 patients. The most frequent metastatic sites were the liver in 22 patients, lung in 6, peritoneum/carcinomatosis in 7, extra-regional lymph nodes in 5, and ovary in 2. Some patients had more than one metastatic site. These patients were managed within a multidisciplinary curative-intent or conversion strategy, including systemic treatment and, when feasible, resection or local treatment of metastatic disease.

To provide an overview of the institutional EOCRC cohort, baseline, surgical, pathological, and recurrence-related outcomes are initially reported for the overall population. A dedicated colon-versus-rectum analysis is then presented to account for the relevant anatomical, therapeutic, and oncological differences between these two entities.

Comprehensive molecular profiling was available for all patients, as tumor molecular testing is routinely implemented at our institution.

Most procedures were performed in an elective setting (71 patients, 80.7%), while 17 patients (19.3%) underwent emergency surgery. The surgical approach was laparoscopic in 46 patients (52.3%), robotic in 25 (28.4%), and open in 17 (19.3%).

Stoma formation was performed in 23 patients (26.1%) and was more frequent in rectal cancer compared to colon cancer (58.6% vs. 10.2%, *p* < 0.001). The median operative time was 233 min [IQR 200.0–271.8], and the median length of hospital stay was 5 days [IQR 3.0–7.2].

Postoperative complications within 90 days occurred in 17 patients (19.3%). According to the Clavien–Dindo classification during index hospitalization, complications were grade I in 6 patients (6.7%), grade II in 19 (21.6%), grade IIIa in 3 (3.4%), grade IIIb in 6 (6.8%), and grade V in 1 (1.1%).

R0 resection was achieved in all patients (Table 3). The median number of lymph nodes harvested was 25 [IQR 17.0–35.2], and the median number of positive lymph nodes was 0 [IQR 0.0–3.0]. Pathological nodal involvement (pN+) was observed in 42 patients (47.7%).

CEA was above the standard reference value (>3.5 ng/mL) in 38 patients (43.2%), while CA19-9 was elevated (>37 U/mL) in 22 patients.

Perineural invasion was present in 55 patients (62.5%), tumor budding in 15 (17.0%), and tumor deposits in 14 (15.9%).

Regarding molecular profile, 79 tumors (89.8%) were microsatellite stable. Mismatch repair (MMR) status was assessed by immunohistochemistry for MLH1, MSH2, MSH6, and PMS2 in all patients, according to institutional protocols. MSH2 loss was identified in 4 patients (4.5%), MSH6 loss in 2 (2.3%), MLH1 loss in 5 (5.7%) and PMS2 in 4 (4.5%). Extended molecular profiling was performed only in selected cases according to clinical indication and multidisciplinary decision-making. KRAS mutation was identified in 2 patients and NRAS mutation in 1 patient. BRAF status was not included in the present analysis; therefore, no BRAF-related results are reported (Table 3).

Adjuvant therapy was planned in 63 patients (71.6%) and initiated in 64 (72.7%). During follow-up, recurrence occurred in 40 patients (45.5%). The pattern of recurrence was predominantly distant, accounting for 30 cases (75.0%), whereas locoregional recurrence was less frequent (22.5%) and isolated local recurrence was rare (2.5%) (Table 4).

As shown in Table 5, among patients who developed recurrence 40 (45.5%), 39 (44.3%) received systemic chemotherapy and 1 (1.1%) radiotherapy.

Among the 40 patients with recurrence, the most frequent recurrence sites were lung in 13 patients, liver in 11, peritoneum/carcinomatosis in 10, nodal disease in 7, bone in 2, and local/pelvic/anastomotic recurrence in 1. Some patients had more than one recurrence site; therefore, site-specific categories were not mutually exclusive.

Patients who developed recurrence, 21 (52.5%) underwent surgical reintervention (Table 5). Most reinterventions were performed for oligometastatic disease, with hepatic (28.6%) and pulmonary resections (23.8%) representing the most common procedures. Less frequently, patients underwent cytoreductive surgery with HIPEC or major abdominal procedures. Despite the low rate of isolated local recurrence, one patient underwent colorectal resection for local relapse. Two patients (9.5%) were found to be unresectable at surgical exploration and received palliative procedures (Table 6).

### 3.2. Colon Versus Rectal Cancer

Clinicopathological characteristics were compared between colon and rectal cancers (Table 7).

Rectal tumors represented the most frequent single anatomical location. Patients with colon cancer (*n* = 59) had a median age of 43.0 years [IQR 39.0–45.8], while those with rectal cancer (*n* = 29) had a median age of 46.0 years [IQR 43.0–48.0].

Median CEA levels were 3.4 [IQR 1.0–13.4] in colon cancer and 2.5 [IQR 1.2–3.9] in rectal cancer. Median CA 19-9 levels were 14.2 [IQR 4.0–46.0] and 8.9 [IQR 5.9–9.8], respectively. Median length of stay was 5.0 days in both groups.

Postoperative morbidity differed between groups. Overall complications were more frequent after rectal cancer surgery compared to colon cancer (27.6% vs. 13.6%), although this difference did not reach statistical significance (*p* = 0.11). However, the distribution of complications according to Clavien–Dindo classification showed a clear difference in severity. While all complications observed after colon surgery were minor (grade I–II), rectal cancer surgery was associated with a higher proportion of major complications (grade ≥III), including one postoperative death (*p* < 0.001) (Table 8).

The median number of lymph nodes harvested was 28.5 [IQR 21.0–43.0] in colon cancer and 17.0 [IQR 12.0–25.0] in rectal cancer. The median number of positive lymph nodes was 1.0 [IQR 0.0–4.0] and 0.0 [IQR 0.0–1.0], respectively.

### 3.3. Age Subgroup Analysis

Age subgroup analysis is detailed in Table 9. Patients younger than 40 years had a higher lymph node harvest and showed differences in MMR profile compared with patients aged 40–50 years, while no relevant differences were observed in postoperative outcomes, stoma formation, or recurrence-related surgical management.

Median CEA levels were 2.4 [IQR 1.1–12.3] in the <40 group and 2.9 [IQR 1.1–10.4] in the 40–50 group. Median CA 19-9 levels were 39.9 [IQR 27.4–79.0] and 8.0 [IQR 4.2–27.7], respectively.

The median number of lymph nodes harvested was 34.0 [IQR 25.2–43.8] in patients younger than 40 years and 24.0 [IQR 16.5–32.5] in those aged 40–50 years. The median number of positive lymph nodes was 1.0 [IQR 0.0–2.8] and 0.0 [IQR 0.0–3.0], respectively.

Perineural invasion was present in 15 patients (60%) in the <40 group and 38 (60.3%) in the 40–50 group. Tumor budding was observed in 3 patients (12%) and 12 (19.0%), while tumor deposits were identified in 1 (4.0%) and 12 (19.0%), respectively.

### 3.4. Surgical Approach and Outcomes

Outcomes according to surgical approach are summarized in Supplementary Table S2. No inferential comparisons were performed because of the limited sample size and descriptive intent of the analysis.

Postoperative complications within 90 days occurred in 5 patients (29.4%) in the open group, 8 (17.4%) in the laparoscopic group, and 4 (16.0%) in the robotic group. Clavien–Dindo grade ≥III complications were reported in 1 patient (5.9%) in the open group and none in the minimally invasive groups.

The median length of hospital stay was 10.0 days [IQR 6.0–15.0] in the open group, 4.0 days [IQR 3.0–7.0] in the laparoscopic group, and 4.0 days [IQR 3.0–5.0] in the robotic group.

Median operative time was 240.0 min [IQR 200.0–300.0] for open surgery, 205.0 min [IQR 180.0–265.0] for laparoscopic surgery, and 240.0 min [IQR 230.0–270.0] for robotic surgery.

## 4. Discussion

The present study provides a real-world description of young patients with colorectal cancer treated with curative intent in a tertiary referral center. Although early-onset colorectal cancer is increasingly recognized as a distinct clinical problem, many aspects of its presentation, biological behavior, and optimal management remain incompletely defined. In this context, our series adds institutional data on a cohort characterized by a relevant burden of advanced disease and adverse pathological features. Recurrence was frequent and predominantly distant, involving mainly the lung, liver, peritoneum, and lymph nodes. At the same time, more than half of the patients who developed recurrence underwent further surgical treatment, suggesting that selected patients may still benefit from an active multidisciplinary strategy when recurrent disease remains potentially treatable.

In this cohort of patients with EOCRC, a substantial proportion presented with advanced pathological features at the time of surgery. In detail, 47.7% of patients demonstrated pathological lymph node involvement (pN+), a proportion consistent with large international series reporting nodal positivity rates ranging from 45% to 55% among young patients [1,4,8]. These findings are consistent with current evidence supporting the safety and effectiveness of minimally invasive approaches in colorectal cancer surgery [1].

In addition to nodal involvement, a high prevalence of adverse histopathological features was observed, including perineural invasion (62.5%), tumor budding (17.0%), and tumor deposits (15.9%). These features are well-established prognostic factors in colorectal cancer and appear to be frequently present in younger populations [6,8,9,15]. Their distribution in this cohort supports the observation that EOCRC may present with biologically aggressive characteristics [27].

In our cohort, recurrence was predominantly distant, consistent with the aggressive biological behavior described in EOCRC. However, more than half of the patients with recurrence underwent surgical reintervention, mainly for liver and lung metastases. This suggests that a relevant proportion of recurrences may still be amenable to a curative-intent surgical approach in selected patients, highlighting the importance of multidisciplinary evaluation.

Despite these unfavorable pathological findings, surgical outcomes were consistent with established oncological quality standards. All resections achieved R0 status, and the median lymph node harvest was above commonly accepted thresholds. These results likely reflect the impact of treatment in a high-volume tertiary referral center and suggest that adequate oncological resection can be consistently achieved in this patient population [8,9].

Minimally invasive surgery was performed in the majority of patients and was associated with a shorter length of hospital stay, without an increase in postoperative morbidity. These findings are in line with current evidence supporting the safety and effectiveness of laparoscopic and robotic approaches in colorectal cancer, including in younger and potentially more advanced-stage patients [8].

The higher rate of stoma formation in rectal cancer likely reflects the greater technical complexity of low pelvic surgery and the frequent need for diversion to protect distal anastomoses. In line with this, rectal cancer surgery was also associated with a higher proportion of major postoperative complications, further supporting the increased surgical complexity of these procedures.

Although overall complication rates were not significantly different (*p*-value = 0.11), rectal cancer surgery was associated with a higher proportion of major complications (27.6%).

The analysis according to age subgroups revealed differences in molecular profile, with a higher proportion of mismatch repair alterations observed in patients younger than 40 years. Although the sample size is limited, this finding is consistent with the known contribution of hereditary cancer syndromes in EOCRC and supports the role of systematic molecular assessment in younger patients [6,8,9,28].

The increasing incidence of EOCRC—now accounting for approximately 10% of newly diagnosed colorectal cancers in high-income countries [3,6,29,30]—together with the proportion of cases associated with hereditary or germline alterations [6,9,28], highlights the importance of early recognition and appropriate referral for genetic counseling in this population.

Taken together, these findings suggest that EOCRC in a tertiary referral setting is often associated with advanced pathological features and systemic recurrence, but also that recurrent disease may remain treatable in a selected proportion of patients. This balance between unfavorable disease presentation and potential therapeutic opportunities represents one of the main clinical messages of the present series. The present study provides a comprehensive assessment of EOCRC within a single high-volume center. Strengths include the inclusion of consecutive patients, detailed clinicopathological and molecular characterization, and standardized reporting of surgical outcomes. Subgroup analyses were also performed to explore differences according to tumor location, age, and surgical approach.

Several limitations should be acknowledged. First, the retrospective single-center design may limit generalizability and introduce potential selection bias. Moreover, although consecutive patients were included, the cohort was not population-based and reflects the activity of a high-volume tertiary referral center; therefore, referral patterns, institutional expertise, and case-mix complexity may have influenced the characteristics and outcomes observed. Second, the relatively modest sample size reduces the ability to detect small differences between subgroups, particularly in molecular analyses. Third, long-term oncological outcomes were not uniformly available, as follow-up duration varied across the study period (median 31.3 months [IQR 21.6–44.6]). In addition, the proportion of patients referred from other hospitals could not be reliably assessed, as referral origin was not systematically recorded in the database used for the present analysis. Extended molecular profiling was not systematically available for all patients. KRAS and NRAS alterations were available only in selected cases, and BRAF status was not included in the present analysis. Therefore, molecular results beyond MMR status should be interpreted with caution.

Further multicenter studies with larger cohorts and longer follow-up are needed to better define the biological behavior of EOCRC and to optimize management strategies in this growing patient population.

## 5. Conclusions

Early-onset colorectal cancer is characterized by a high prevalence of adverse pathological features and a substantial rate of distant recurrence. However, a significant proportion of recurrences remain amenable to surgical reintervention, particularly in selected patients with oligometastatic disease.

Management in a high-volume tertiary center allows achievement of optimal surgical quality metrics and supports the safe use of minimally invasive approaches. These findings highlight the importance of a multidisciplinary strategy and reinforce the role of an aggressive surgical approach in selected EOCRC patients.

## Figures and Tables

**Table 1:** Demographic characteristics of the overall cohort.

Variable	Overall Cohort *n* = 88
Age at surgery (years)	44.0 [39.8–47.0]
Sex	
Male	45 (51.1%)
Female	43 (48.9%)
Tumor location	
Right colon	19 (21.8%)
Transverse colon	5 (5.7%)
Splenic flexure	2 (2.3%)
Left colon	10 (11.5%)
Sigmoid colon	23 (26.1%)
Upper rectum	10 (11.5%)
Middle rectum	11 (12.6%)
Lower rectum	8 (9.2%)

Data are presented as median [IQR] or *n* (%).

**Table 2:** Surgical outcomes.

Variable	Overall Cohort (*n* = 88)
Surgical setting	
Elective	71 (80.7%)
Emergency	17 (19.3%)
Surgical approach	
Open	17 (19.3%)
Laparoscopic	46 (52.3%)
Robotic	25 (28.4%)
Stoma formation	
No	65 (73.9%)
Yes	23 (26.1%)
Operative time (min)	233 [200–271.8]
Length of stay (days)	5 [3–7.2]
Clavien–Dindo classification during index hospitalization (highest grade per patient)	
Grade I	6 (6.7%)
Grade II	19 (21.6%)
Grade IIIb	6 (6.8%)
Grade IIIa	3 (3.4%)
Grade V	1 (1.1%)
Complications after discharge within 90 days	
No	71 (81.8%)
Yes	17 (19.2%)

**Table 3:** Pathological and oncological outcomes.

Variable	Overall Cohort (*n* = 88)
Resection margins (R)	
R0	88 (100.0%)
Lymph nodes harvested (*n*)	25.0 [17.0–35.2]
Positive lymph nodes (*n*)	0.0 [0.0–3.0]
Patients with Nodal Involvement (pN+)	42 (47.7%)
Perineural invasion	
Yes	55 (62.5%)
No	33 (37.5%)
Tumor budding	
No	73 (83.0%)
Yes	15 (17.0%)
Tumor deposits	
No	74 (84.1%)
Yes	14 (15.9%)
Mismatch repair status	
pMMR/MSS	79 (89.8%)
MSH2 loss	4 (4.5%)
MSH6 loss	2 (2.3%)
MLH1 loss	5 (5.7%)
PMS2 loss	4 (4.5%)
RAS mutation status	
KRAS mutation	2
NRAS mutation	1

Protein-loss categories are not mutually exclusive, as some tumors showed combined loss of more than one MMR protein.

**Table 4:** Pattern of recurrence in the overall cohort (*n* = 88).

Recurrence Pattern	*n* (%) of Recurrences
Local recurrence	1 (2.5%)
Locoregional recurrence	9 (22.5%)
Distant recurrence *	30 (75.0%)
Total recurrences in cohort	40/88 (45.5%)

* Distant recurrence included liver, lung, peritoneal, distant nodal, bone, brain, or other extra-regional disease. Peritoneal recurrence was classified as distant recurrence. Patients with combined patterns of recurrence, including local/locoregional and distant disease, were classified as distant recurrence.

**Table 5:** Treatment of recurrence.

Treatment	*n* (%)
Systemic chemotherapy	39 (97.5%)
Radiotherapy	1 (2.5%)

**Table 6:** Surgical reintervention in patients with recurrence (*n* = 40).

Category of Reintervention	*n*	% of Reoperated Patients (*n* = 21)	% of Total Recurrences (*n* = 40)
Hepatic resection	6	28.6%	15%
Pulmonary resection	5	23.8%	12.5%
Cytoreductive surgery + HIPEC	2	9.5%	5.0%
Major abdominal surgery (total colectomy, pelvic exenteration)	2	9.5%	5.0%
Gynecological surgery	2	9.5%	5.0%
Colorectal resection (local recurrence)	1	4.8%	2.5%
Palliative/unresectable at exploration	2	9.5%	5.0%
Liver transplantation (other centre)	1	4.8%	2.5%
Total reoperated patients	21	100%	52.5%

The patient with isolated local recurrence underwent colorectal resection with anastomosis. One patient was deemed unresectable intraoperatively and was classified within the palliative group.

**Table 7:** Comparison between colon and rectal cancer.

Variable	Colon (*n* = 59)	Rectum (*n* = 29)	*p*-Value
Age (years)	43.0 [39.0–45.8]	46.0 [43.0–48.0]	
CEA	3.4 [1.0–13.4]	2.5 [1.2–3.9]	
CA 19-9	14.2 [4.0–46.0]	8.9 [5.9–9.8]	0.619
Length of stay (days)	5.0 [3.0–7.0]	5.0 [3.0–8.0]	
Lymph nodes harvested (*n*)	28.5 [21.0–43.0]	17.0 [12.0–25.0]	
Stoma formation, *n* (%)	6 (10.2%)	17 (58.6%)	<0.001
Positive lymph nodes (*n*)	1.0 [0.0–4.0]	0.0 [0.0–1.0]	
Mismatch repair status			0.654
pMMR/MSS	51 (86.4%)	28 (93.1%)	
MSH2 loss	4 (6.8%)	1 (3.5%)	
MSH6 loss	2 (3.4%)	0 (0.0%)	
MLH1 loss	4 (6.8%)	1 (3.5%)	
PMS2 loss	3 (5.1%)	1 (3.5%)	
RAS mutations status			
KRAS mutation	1	1	
NRAS mutation	1	0	

**Table 8:** Postoperative morbidity according to tumor location and Clavien–Dindo classification.

	Colon (*n* = 59)	Rectum (*n* = 29)	*p*-Value
Any complication, *n* (%)	8 (13.6%)	8 (27.6%)	0.11
Clavien–Dindo grade, *n* (%)			
Grade I–II	8 (13.6%)	1 (3.4%)	
Grade ≥ III	0 (0%)	8 (27.6%)	<0.001

*p*-values were calculated using chi-square test for overall morbidity and Fisher’s exact test for major complications (Clavien–Dindo ≥III).

**Table 9:** Age subgroup analysis.

Variable	<40 (*n* = 25)	40–50 (*n* = 63)	*p*-Value
Age (years)	33.5 [32.0–37.0]	45.0 [43.0–48.0]	<0.001
CEA	2.4 [1.1–12.3]	2.9 [1.1–10.4]	0.872
CA 19-9	39.9 [27.4–79.0]	8.0 [4.2–27.7]	0.093
Length of stay (days)	5.0 [4.0–7.5]	5.0 [3.0–7.0]	0.687
Lymph nodes harvested (*n*)	34.0 [25.2–43.8]	24.0 [16.5–32.5]	0.012
Positive lymph nodes (*n*)	1.0 [0.0–2.8]	0.0 [0.0–3.0]	0.478
Female sex	11 (44.0%)	32 (50.8%)	1.000
Emergency surgery	6 (24.0%)	11 (17.5%)	0.360
Minimally invasive approach (laparoscopic/robotic)	21 (84.0%)	50 (79.4%)	1.000
Stoma formation	4 (16.0%)	19 (30.1%)	0.172
90-day complications	3 (12.0%)	13 (20.6%)	0.546
Clavien–Dindo ≥III	1 (4.0%)	9 (10.2%)	0.259
Perineural invasion	15 (60.0%)	38 (60.3%)	0.613
Tumor budding	3 (12.0%)	12 (19.0%)	0.750
Tumor deposits	1 (4.0%)	12 (19.0%)	0.169
Mismatch repair status			0.035
pMMR/MSS	20 (84%)	59 (93.7%)	
MSH2 loss	2 (8.0%)	2 (3.2%)	
MSH6 loss	0 (0.0%)	2 (3.2%)	
MLH1 loss	3 (12.0%)	2 (3.2%)	
PMS2 loss	3 (12.0%)	1 (1.6%)	
RAS mutation status			
NRAS mutation	1	1	
KRAS mutation	0	1	

## Data Availability

The data presented in this study are available from the corresponding author upon reasonable request.

## Supplementary Materials

The following supporting information can be downloaded at: https://www.mdpi.com/article/10.3390/cancers18121934/s1. Table S1: STROBE checklist for cohort studies. Table S2: Outcomes according to surgical approach.

## Abbreviations

EOCRC	Early-Onset Colorectal Cancer
CRC	Colorectal Cancer
LOS	Length of Stay
IQR	Interquartile Range
SD	Standard Deviation
CEA	Carcinoembryonic Antigen
CA 19-9	Carbohydrate Antigen 19-9
MMR	Mismatch Repair
MSS	Microsatellite Stable
