# Supplementary material for: Early-Onset Colorectal Cancer: Clinicopathological Features and Surgical Outcomes in Patients Treated with Curative Intent at a Tertiary Center
Source: Cancers (Basel). 2026 Jun 14;18(12):1934. doi: 10.3390/cancers18121934 (PMC13296778; doi:10.3390/cancers18121934)
Supplement: Supplementary file 1 [file cancers-18-01934-s001.zip › Supplementary Table S2.pdf]

Supplementary Table S2. Outcomes according to surgical approach.

Approach	N	90-day complications	Clavien ⩾III	Length of stay (median [IQR])	Operative time (median [IQR])
Open	17	5/17 (29.4%)	1/17 (5.9%)	10.0 [6.0–15.0]	240.0 [200.0–300.0]
Laparoscopic	46	8/46 (17.4%)	0/46 (0.0%)	4.0 [3.0–7.0]	205.0 [180.0–265.0]
Robotic	25	4/25 (16.0%)	0/25 (0.0%)	4.0 [3.0–5.0]	240.0 [230.0–270.0]

** no inferential comparisons were made by sample size or by descriptive intent.*
